# Supplementary material for: The Modulation of SCO2730/31 Copper Chaperone/Transporter Orthologue Expression Enhances Secondary Metabolism in Streptomycetes
Source: Int J Mol Sci. 2021 Sep 20;22(18):10143. doi: 10.3390/ijms221810143 (PMC8464937; doi:10.3390/ijms221810143)
Supplement: Supplementary file 1 [file ijms-22-10143-s001.zip › Table S3.pdf]

Table S3. Bacterial strains, plasmids and primers used in this study.

Strain	Description	Reference
S.coelicolor M145	SCP1- SCP2⁻ , reference strain.	[1]
Streptomyces venezuelae NRRL B-65442	NRRL B-65442 strain	
SCO2730::Tn5062 mutant	*S. coelicolor* interrupted at the *SCO2730* ORF	[2]
Streptomyces albus J1074	*S.albus* G lacking *SalI* restriction modification system	[3]
E. coli TOP10	F^- *mcr*A Δ (*mrr-hsd*RMS-*mcr*BC) ϕ80*lac*ZΔM15 Δ*lac*X74 *rec*A1 *ara*D139 Δ (*ara-leu*)7697 *gal*U *gal*K *rps*L *end*A1 *nup*G.	Invitrogen
E. coli ET12567/pUZ8002	*E. coli* ET12567 harbouring pUZ8002, a not self-transmissible plasmid which can mobilize *oriT*-containing plasmids by conjugation.	[4]

**Plasmids**		
pNG4	Integrative and conjugative vector, $Hygro^R$.	[5]
pNG4-SP44-*SCO2730/31* *S. coelicolor*	pNG4 harbouring the *S. coelicolor SCO2730/31* antisense mRNA under the SP44 promoter control	This study
pNG4-SP44-*SCO2730/31* consensus	pNG4 harbouring the consensus *SCO2730/31* antisense mRNA under the SP44 promoter control	This study
PCR™-Blunt II-TOPO®	Zero Blunt® TOPO® PCR Cloning Kit, Kan^R.	Invitrogen
pKQLL-SP44-*SCO2730/31* *S. coelicolor*	Kan^R, pBR322 replication origin, M13F/R harbouring the *S. coelicolor SCO2730/31* antisense mRNA under the SP44 promoter control	BGI, this study
pKQLL-SP44-*SCO2730/31* consensus	Kan^R, pBR322 replication origin, M13F/R harbouring the consensus *SCO2730/31* antisense mRNA under the SP44 promoter control	BGI, this study

**Primers**		
SCO4848F	CGTCGTATCCCCTCGGTTG	[6]
pMS82R	GAGCCGGGAAAGCTCATTCA	[6]
vnz22340	TTACTTCTTGCTTGCTCGGAC	This study
M13F	CAGGAAACAGCTATGA	Invitrogen
M13R	CTGGCCGTCGTTTTAC	Invitrogen
SP44F	TCTCACTCCGCTGAAACTGT	This study
SP44R	ATGCTAGTCGCGGTTGATCG	This study

References

1. Kieser, T., *Practical streptomyces genetics*. John Innes Foundation: Norwich, 2000.
2. Gonzalez-Quinonez, N.; Corte-Rodriguez, M.; Alvarez-Fernandez-Garcia, R.; Rioseras, B.; Lopez-Garcia, M. T.; Fernandez-Garcia, G.; Montes-Bayon, M.; Manteca, A.; Yague, P., Cytosolic copper is a major modulator of germination, development and secondary metabolism in Streptomyces coelicolor. *Sci Rep* **2019,** 9, (1), 4214.
3. Chater, K. F.; Wilde, L. C., Streptomyces albus G mutants defective in the SalGI restriction-modification system. *J Gen Microbiol* **1980,** 116, (2), 323-34.
4. Flett, F.; Mersinias, V.; Smith, C. P., High efficiency intergeneric conjugal transfer of plasmid DNA from Escherichia coli to methyl DNA-restricting streptomycetes. *FEMS Microbiol Lett* **1997,** 155, (2), 223-9.
5. Gonzalez-Quinonez, N.; Lopez-Garcia, M. T.; Yague, P.; Rioseras, B.; Pisciotta, A.; Alduina, R.; Manteca, A., New PhiBT1 site-specific integrative vectors with neutral phenotype in Streptomyces. *Appl Microbiol Biotechnol* **2016,** 100, (6), 2797-808.
6. Rioseras, B.; Yague, P.; Lopez-Garcia, M. T.; Gonzalez-Quinonez, N.; Binda, E.; Marinelli, F.; Manteca, A., Characterization of SCO4439, a D-alanyl-D-alanine carboxypeptidase involved in spore cell wall maturation, resistance, and germination in Streptomyces coelicolor. *Sci Rep* **2016,** 6, 21659.
